# Preparation and Thermoelectric Performance of Non-Stoichiometric Skinnerite

**DOI:** 10.3390/ma18102372

**Published:** 2025-05-20

**Authors:** Sang Jun Park, Il-Ho Kim

**Affiliations:** Department of Materials Science and Engineering, College of Engineering, Korea National University of Transportation, Chungju 27469, Republic of Korea; psj8479@naver.com

**Keywords:** thermoelectric, skinnerite, non-stoichiometric, off stoichiometric

## Abstract

Non-stoichiometric skinnerite Cu_3+m_SbS_3_ (−0.04 ≤ m ≤ 0.04) was synthesized via mechanical alloying and hot pressing. A phase analysis, microstructural characterization, and thermoelectric property evaluation were conducted to investigate the effects of Cu deficiency and excess. An X-ray diffraction of the mechanically alloyed powders confirmed the formation of cubic skinnerite, while the hot-pressed Cu-rich samples contained a secondary phase, identified as cubic tetrahedrite (Cu_12_Sb_4_S_13_). The lattice constant decreased within the range of 1.0341–1.0347 nm for the non-stoichiometric compositions. The microstructural analysis revealed a skinnerite matrix with tetrahedrite inclusions in the Cu-excess samples. The differential scanning calorimetry showed a single endothermic peak at 876 K for the stoichiometric skinnerite, corresponding to its melting point, whereas the non-stoichiometric samples exhibited additional phase transitions at 814–818 K, and a melting reaction at 873–874 K. The electrical conductivity increased with the temperature, indicating non-degenerate semiconductor behavior. Between 323 and 423 K, the electrical conductivity varied depending on the Cu deficiency or excess, but above 423 K, all the non-stoichiometric samples exhibited higher electrical conductivity than the stoichiometric skinnerite. A positive Seebeck coefficient confirmed p-type conduction in all the samples, while Cu deficiency led to a decrease in the Seebeck coefficient but enhanced the power factor, due to increased electrical conductivity. The Cu_2.98_SbS_3_ sample exhibited the highest power factor of 0.85 mWm^−1^K^−2^ at 623 K. Although Cu deficiency resulted in increased thermal conductivity due to a higher carrier concentration, the significant enhancement in the power factor led to a maximum dimensionless figure of merit (ZT) of 0.59 at 623 K for Cu_2.98_SbS_3_, surpassing the ZT of 0.51 for the stoichiometric Cu_3_SbS_3_.

## 1. Introduction

Thermoelectric technology is anticipated to play a critical role in enhancing energy efficiency by recovering waste heat and directly converting it into electricity [1,2,3,4,5,6]. The thermoelectric conversion efficiency is characterized by the figure of merit (Z), defined as Z = PF/κ = α^2^σ/(κ_L_ + κ_E_), where PF is the power factor, α is the Seebeck coefficient, σ is the electrical conductivity, and κ is the thermal conductivity, comprising the lattice thermal conductivity (κ_L_) and electronic thermal conductivity (κ_E_) [7]. Since these parameters are temperature-dependent, a dimensionless figure of merit, ZT, is used, which is obtained by multiplying the figure of merit by the absolute temperature (T).

Cu–Sb–S compounds, including Cu_12_Sb_4_S_13_, CuSbS_2_, Cu_3_SbS_3_, and Cu_3_SbS_4_, offer significant advantages over conventional thermoelectric materials due to their low toxicity, earth abundance, and cost-effectiveness. Among these, Cu_3_SbS_3_ (skinnerite) exhibits the lowest lattice thermal conductivity, attributed to its large S–Sb–S bond angle [8]. Additionally, its high absorption coefficient, p-type electrical conductivity, and an optical bandgap of 1.5 eV make it a promising candidate for solar cell applications [9]. However, studies on the thermoelectric properties of Cu_3_SbS_3_ remain relatively limited. Cu_3_SbS_3_ undergoes three structural phase transitions: an orthorhombic phase (space group P2_1_2_1_2_1_) below 263 K, a monoclinic phase (P2_1_/c) between 263 and 395 K, and another orthorhombic phase (Pnma) above 395 K [10,11]. As the temperature increases, the copper atoms become increasingly disordered. Furthermore, Cu_3_SbS_3_ can adopt a cubic structure, resembling that of tetrahedrite (Cu_12_Sb_4_S_13_ or Cu_3_SbS_3.25_) [12]. Jiasong et al. [13] synthesized cubic Cu_3_SbS_3_ nanorods using a solvothermal method, while Maiello et al. [14] fabricated cubic Cu_3_SbS_3_ thin films via magnetron sputtering. In our previous studies [15,16], we successfully synthesized a stable cubic skinnerite Cu_3_SbS_3_ phase between 323 and 623 K, reporting a maximum ZT of 0.51 at 623 K.

Recent studies have investigated the charge transport and thermoelectric properties of non-stoichiometric Cu–chalcogenide compounds. Jung et al. [17] enhanced the power factor of famatinite (Cu_3_SbS_4_) by increasing the carrier concentration through a Cu deficiency; however, this also elevated the thermal conductivity, resulting in a ZT similar to that of stoichiometric famatinite. Conversely, Kumar et al. [18] reported an improved ZT by simultaneously increasing the carrier concentration and reducing the thermal conductivity via a Cu deficiency. Kim and Kim [19] and Wei et al. [20] achieved the superior thermoelectric performance of permingeatite (Cu_3_SbSe_4_) by enhancing the carrier concentration and power factor through a Cu deficiency. Do and Mahanti [21] showed that Cu vacancies in permingeatite are energetically favorable and generate holes, leading to p-type conduction. Tsujii et al. [2,22,23] reported that a Cu deficiency and Fe excess in chalcopyrite (CuFeS_2_) decreased both the electrical resistivity and thermal conductivity, increasing the power factor fivefold and the ZT tenfold. Li et al. [24] and Xie et al. [25] improved the ZT of chalcopyrite by inducing a sulfur deficiency or excess, with the latter forming mixed phases (Cu_1.1_Fe_1.1_S_2_ and CuFeS_2_) that enhanced both the electrical and thermal transport. Zhai et al. [26] observed an increased power factor and reduced thermal conductivity in Cu-deficient eskebornite (CuSbSe_2_), while Chen et al. [27] reported a 44% increase in the ZT of pribramite (CuFeSe_2_) due to an Sb deficiency. Kwak and Kim [28] found that Cu excess in tetrahedrite (Cu_12_Sb_4_S_13_) reduced the thermal conductivity via phonon scattering, whereas Cu deficiency enhanced the thermoelectric performance by increasing the hole carriers.

In this study, non-stoichiometric skinnerite Cu_3+m_SbS_3_ was synthesized to investigate the effects of the Cu content on the thermoelectric performance. Limited experimental data exist on the thermoelectric properties of skinnerite, and to the best of our knowledge, no prior studies have reported on non-stoichiometric Cu_3_SbS_3_. Given the promising enhancements in the thermoelectric performance observed in non-stoichiometric Cu–chalcogenide compounds, this study aimed to explore the potential of Cu-deficient and Cu-excess skinnerite as thermoelectric materials.

## 2. Experimental Procedure

High-purity elemental powders of Cu (purity 99.9%, <45 μm), Sb (purity 99.999%, <150 μm), and S (purity 99.99%, <75 μm) were used to synthesize Cu_3+m_SbS_3_ (m = −0.04, −0.02, 0, 0.02, and 0.04) via mechanical alloying (MA). The precursor powders, weighed according to their nominal composition, were loaded into a stainless-steel vial along with 5 mm diameter stainless-steel balls. To prevent oxidation, the vial was purged with Ar gas before sealing, and MA was performed at a rotational speed of 350 rpm for 18 h. The resulting powder was consolidated via hot pressing (HP) at 623 K under a uniaxial pressure of 70 MPa for 2 h in a vacuum using a graphite mold with a 10 mm internal diameter. The optimized MA–HP conditions for the synthesis and densification of skinnerite were established in our previous studies [15,16].

Phase identification of the synthesized powders and sintered specimens was conducted using X-ray diffraction (XRD; D8-Advance, Bruker, Billerica, MA, USA) with Cu Kα radiation. Lattice parameters were determined via Rietveld refinement using TOPAS software (v4.1, Bruker, Billerica, MA, USA). The microstructure was examined using scanning electron microscopy (SEM; Prisma E, Thermo Fisher Scientific, Waltham, MA, USA) in backscattered electron (BSE) mode. Elemental distribution and phase composition analyses were performed using energy-dispersive X-ray spectroscopy (EDS; Quantax200, Bruker) for both one- and two-dimensional mapping. Thermal analysis was conducted using thermogravimetric and differential scanning calorimetry (TG–DSC; TGA/DSC1, Mettler Toledo, Columbus, OH, USA) in the temperature range of 300–950 K, with a heating rate of 5 Kmin^−1^.

The van der Pauw method was employed to evaluate the charge transport properties, including carrier concentration and mobility, using a data acquisition system (TC2110, Keithley Instruments, Solon, OH, USA) integrated with a Hall interface (7065, Keithley Instruments, Solon, OH, USA) and an electromagnet (HV4H, Walker Scientific, Worcester, MA, USA). Electrical conductivity and the Seebeck coefficient were determined via the DC four-probe method using a ZEM-3 system (Advance Riko, Yokohama, Japan). Thermal diffusivity was measured using the laser flash method with a TC-9000H system (Advance Riko, Yokohama, Japan), and thermal conductivity was calculated from the measured specific heat and density of the specimens. The thermoelectric properties, including the power factor (PF) and dimensionless figure of merit (ZT), were evaluated based on electrical conductivity, Seebeck coefficient, and thermal conductivity measurements over a temperature range of 323–623 K.

## 3. Results and Discussion

**Figure 1:**
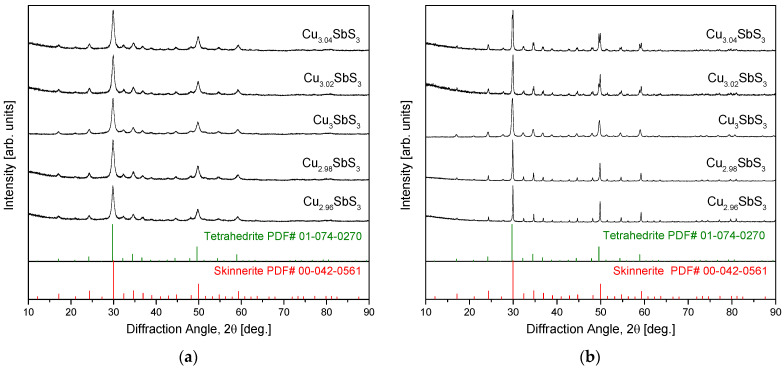
XRD patterns of Cu_3+m_SbS_3_ synthesized using (**a**) MA and prepared via (**b**) MA–HP.

Figure 1a provides the XRD patterns of the Cu_3+m_SbS_3_ powders synthesized via MA, confirming the formation of cubic skinnerite (space group $I\bar{4}3m;$ PDF# 00-042-0561). The absence of diffraction peaks corresponding to monoclinic or orthorhombic skinnerite phases, which were discussed in the introduction, indicates that the cubic phase remained stable under the MA conditions used. This observation aligns with those of previous studies [15,16], reinforcing that the MA process effectively promotes the formation of the cubic skinnerite structure without detectable polymorphic transitions. Figure 1b presents the XRD patterns of the Cu_3+m_SbS_3_ specimens after HP. The cubic skinnerite phase was retained across all the compositions, demonstrating its thermal stability during the HP process. However, the Cu-rich specimens exhibited additional diffraction peaks corresponding to tetrahedrite (Cu_12_Sb_4_S_13_ or Cu_3_SbS_3.25_; PDF# 02-074-0270), with the peak intensities increasing as the Cu content rose. The formation of tetrahedrite as a secondary phase was reasonable given its structural and compositional similarities to skinnerite. The increased Cu content likely stabilized the tetrahedrite within the Cu_3+m_SbS_3_ system, suggesting that the solubility limit of the Cu within the skinnerite phase was exceeded, leading to secondary-phase precipitation.

Interestingly, the behavior of the Cu-deficient specimens differed significantly. Unlike the Cu-rich samples, they did not show any secondary-phase formation, which is consistent with prior studies on the phase stability of non-stoichiometric Cu–chalcogenides. For example, investigations of Cu-deficient permingeatite have reported no phase changes, as confirmed by XRD analyses [18,19,20]. This suggests that skinnerite can accommodate Cu vacancies within its structure without undergoing a structural transformation. Comparable phase-transition behaviors have been observed in related Cu–Sb–S materials. Li et al. [24] found that tetragonal chalcopyrite transitions to cubic chalcopyrite under sulfur-deficient conditions, indicating that an anion deficiency can drive structural changes in similar systems. Additionally, Kwak and Kim [28] demonstrated that in non-stoichiometric tetrahedrite, the lattice constant varied systematically with the Cu content—decreasing with Cu deficiency and increasing with Cu excess—while maintaining a single-phase structure. This suggests that within a certain compositional range, Cu vacancies or excess Cu can be accommodated in the skinnerite or tetrahedrite lattices without triggering complete phase separation.

Table 1 summarizes the lattice constants, relative densities, and phase contents of the Cu_3+m_SbS_3_ specimens synthesized using the MA–HP method. The lattice constant of the stoichiometric Cu_3_SbS_3_ was determined to be 1.0374 nm, aligning with previously reported values. In earlier studies [15,16], the MA powders exhibited a slightly lower lattice constant (1.0343 nm), whereas the HP-processed specimens showed a range of 1.0365–1.0394 nm, suggesting a minor expansion due to the thermal effects during sintering. The Cu-deficient samples in this study exhibited further lattice contraction (1.0346–1.0347 nm), attributed to the Cu vacancies. This phenomenon has been widely observed in non-stoichiometric Cu–chalcogenide compounds, where the removal of Cu resulted in a reduction in both the *a*- and *c*-axis lattice parameters, as reported for tetragonal famatinite [17] and tetragonal permingeatite [19,20]. Interestingly, the cubic tetrahedrite exhibited a different contraction pattern, where only the *a*-axis shrunk with a Cu deficiency. Unexpectedly, the Cu-rich specimens also displayed a reduced lattice constant (1.0341 nm). This anomaly was likely due to the formation of tetrahedrite as a secondary phase, which has a slightly smaller lattice constant (1.0350 nm) than skinnerite [28]. The presence of tetrahedrite thus decreased the overall measured lattice parameter. Kwak and Kim [28] further demonstrated that the lattice constant of non-stoichiometric tetrahedrite varies from 1.0338 to 1.0384 nm, depending on the Cu content, while maintaining a single-phase structure. A similar effect was observed by Zhai et al. [26] in tetragonal eskebornite, where excess Cu led to lattice contraction and the formation of a secondary Cu_2_Se phase. In terms of densification, the stoichiometric skinnerite specimens fabricated via MA–HP achieved a relative density of 99.7%, close to the theoretical density of skinnerite (5.1 gcm^−3^) [29]. The non-stoichiometric skinnerite specimens exhibited slightly lower densities (98.2–99.1%), consistent with the findings of Lee and Kim [16], who reported sintered densities of stoichiometric skinnerite ranging from 94.4% to 99.7%. A Rietveld refinement of the XRD data presented in Figure 1a was used to quantify the phase fractions of the skinnerite and tetrahedrite. The results indicate that higher Cu-excess concentrations promoted a phase transformation from skinnerite to tetrahedrite.

**Figure 2:**
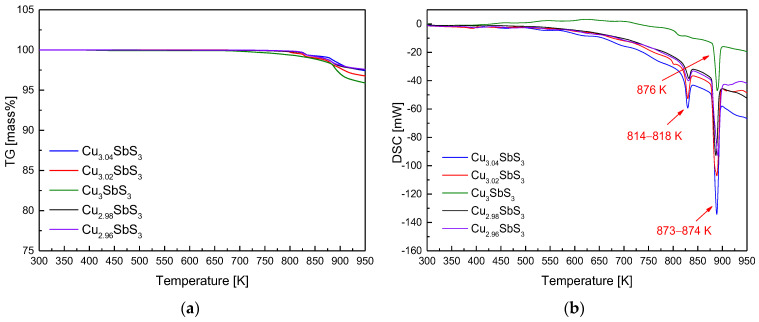
Analyses of (**a**) TG and (**b**) DSC for Cu_3+m_SbS_3_ prepared using the MA–HP process.

Figure 2 provides valuable insights into the thermal stability and phase transitions of the Cu_3+m_SbS_3_ synthesized via the MA–HP method, by analyzing the mass changes and reaction temperatures from 300 to 950 K. The TG analysis in Figure 2a reveals a minor mass loss above 800 K, suggesting volatilization due to phase decomposition, phase transition, or partial melting. This behavior is consistent with the thermal instability of Cu–Sb–S compounds at elevated temperatures, where Cu and S are known to volatilize under certain conditions. The onset of mass loss at this temperature range aligns with previously reported thermal decomposition studies of similar sulfide-based thermoelectric materials. The DSC results in Figure 2b highlight the key thermal events distinguishing the stoichiometric and non-stoichiometric specimens. The single endothermic peak at 876 K in the stoichiometric sample corresponds to the melting point of skinnerite, confirming its thermal stability up to this temperature. However, the non-stoichiometric samples exhibit two distinct endothermic events at 814–818 K and 873–874 K. The lower-temperature peak aligns with the phase transition of tetrahedrite, while the higher-temperature peak corresponds to the melting of skinnerite. These observations are consistent with those of prior studies. Lee and Kim [16] reported similar DSC trends in pure skinnerite, identifying minor endothermic peaks at 798–808 K (phase transition) and major peaks at 865–876 K (melting). Additionally, Skinner et al. [30] noted that tetrahedrite undergoes a phase transition to skinnerite between 795 and 804 K, which supports the interpretation of the lower endothermic peak in the non-stoichiometric samples.

**Figure 3:**
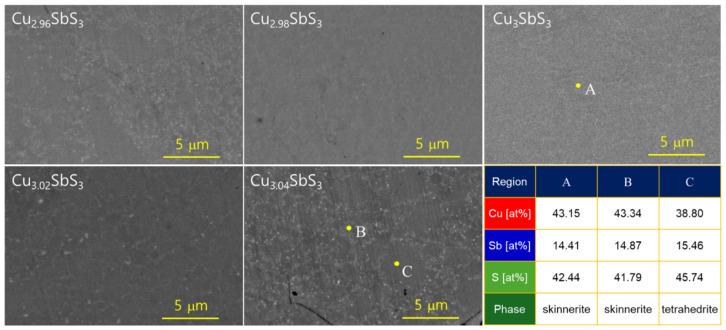
BSE–SEM micrographs and compositional analyses of Cu_3+m_SbS_3_.

Figure 3 displays the BSE-mode SEM images of the Cu_3+m_SbS_3_ sintered samples. The stoichiometric sample exhibited a uniform, single-phase skinnerite microstructure, indicating that the MA–HP process successfully promoted phase purity. This suggests that within the stoichiometric conditions, the Cu_3_SbS_3_ remained thermodynamically stable under the given synthesis parameters. In contrast, the Cu-rich samples revealed the coexistence of skinnerite and tetrahedrite phases. The formation of tetrahedrite (Cu_12_Sb_4_S_13_) in the Cu-rich compositions was expected due to its structural similarity to skinnerite, as both are Cu–Sb–S-based compounds with comparable crystallographic frameworks. The difficulty in distinguishing between these phases in BSE-mode imaging stems from their similar atomic compositions, which result in minimal contrast differences in BSE images. Since BSE contrast primarily depends on the atomic number variations (Z-contrast), the similar mean atomic numbers of skinnerite and tetrahedrite lead to their indistinct appearance. Despite the BSE contrast limitations, the EDS spot analysis provided additional insight into the phase distribution. The higher S/Cu ratio observed in region C strongly suggests the presence of tetrahedrite, as this phase has a slightly different Cu:S stoichiometry compared to skinnerite.

**Figure 4:**
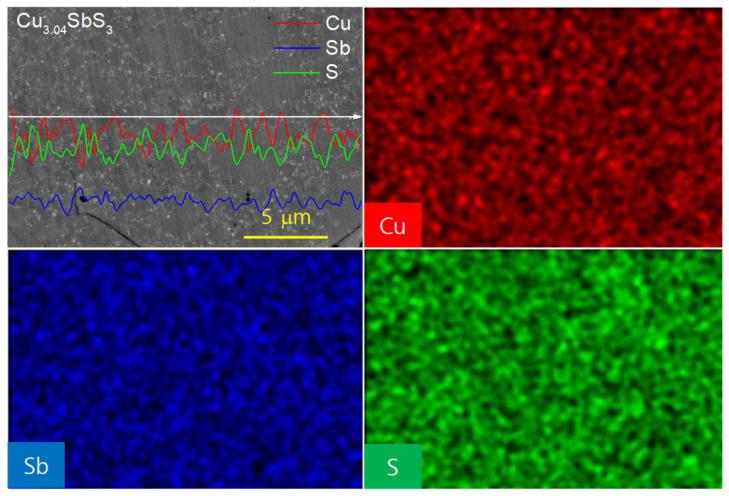
EDS elemental analyses of Cu_3.04_SbS_3_.

Figure 4 provides insight into the elemental distribution and phase segregation of the Cu-excess Cu_3.04_SbS_3_ specimen through EDS elemental line scans and mapping. The concentrations of Cu, Sb, and S exhibit spatial variations across the different regions. A key observation is the inverse correlation between the Cu and S concentrations: the Cu-rich regions exhibit a lower S content, while the S-rich areas contain relatively less Cu. The presence of distinct regions with varying S/Cu ratios supports the coexistence of skinnerite- (Cu_3_SbS_3_) and tetrahedrite-related phases (Cu_12_Sb_4_S_12_ or Cu_3_SbS_3.25_). Tetrahedrite generally accommodates a higher S content than skinnerite, which aligns with the higher S/Cu ratio detected in certain regions. The formation of tetrahedrite in the Cu-excess compositions can be attributed to the material’s intrinsic thermodynamic behavior. Excess Cu may stabilize tetrahedrite-like structures, given that Cu-rich environments favor the formation of Cu–S bonds, which influence phase stability. Additionally, the presence of a Cu-excess phase may impact the carrier concentration and transport properties, as tetrahedrite typically exhibits a lower electrical conductivity than skinnerite due to its more complex crystal structure and different charge carrier scattering mechanisms.

**Figure 5:**
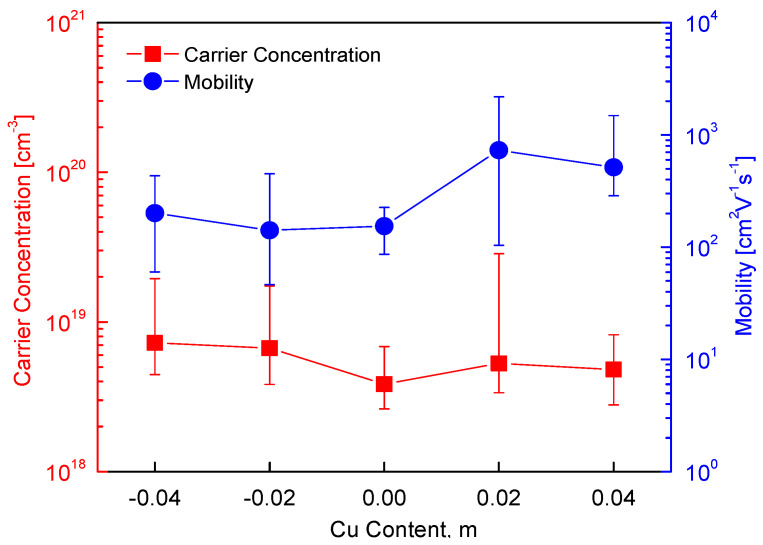
Charge transport properties of Cu_3+m_SbS_3_.

Figure 5 presents the charge transport characteristics of the Cu_3+m_SbS_3_, indicating the effects of the Cu non-stoichiometry on the carrier concentration and mobility. The positive Hall coefficient confirms the p-type conduction of skinnerite (the Hall coefficient data are not plotted in Figure 5), which is attributed to the presence of holes as majority charge carriers, consistent with previous studies [12,16,26]. The carrier concentration and mobility exhibited a dependence on the Cu content, indicating that the point defects influenced the charge transport behavior. In the Cu-deficient specimens, an increase in the hole concentration was observed, which can be attributed to the formation of Cu vacancies. These vacancies acted as acceptor defects, creating additional hole carriers and thereby reinforcing the p-type conductivity. This trend aligns with the general behavior of Cu-based chalcogenides, where Cu vacancies exhibit low formation energy, facilitating their spontaneous generation [31]. Conversely, in the Cu-excess specimens, a reduction in the hole concentration was anticipated due to the potential electron compensation. However, the observed charge transport properties suggest that the formation of a secondary tetrahedrite (Cu_12_Sb_4_S_13_ or Cu_3_SbS_3.25_) phase influenced the carrier concentration. Since tetrahedrite is also a p-type semiconductor with a relatively high carrier concentration (3.12 × 10^20^ cm^−3^) and a low mobility (4.35 cm^2^V^−1^s^−1^) [32], its presence likely modified the charge transport behavior of the Cu_3+m_SbS_3_. The coexistence of skinnerite and tetrahedrite phases introduced a complex charge transport mechanism, where the interface between the phases led to increased carrier scattering, thereby affecting the mobility.

For the stoichiometric Cu_3_SbS_3_, a carrier concentration of 3.84 × 10^18^ cm^−3^ and a mobility of 154 cm^2^V^−1^s^−1^ were obtained, values that are comparable to those of other Cu-based chalcogenides. Previous studies have demonstrated that doping can effectively modulate the charge transport in these materials. For example, Park and Kim [33] reported that Fe doping in Cu_3−y_Fe_y_SbS_3_ (0.02 ≤ y ≤ 0.06) increased the carrier concentration to (1.2–1.7) × 10^19^ cm^−3^ while significantly enhancing the mobility (248–392 cm^2^V^−1^s^−1^), indicating that aliovalent cation substitution can improve the charge transport. Non-stoichiometry in Cu-based chalcogenides has been widely studied, with varying effects on the carrier concentration and mobility. Kumar et al. [18] and Kim and Kim [19] observed that Cu-deficient permingeatite exhibited an increase in carrier concentration but a decrease in mobility due to enhanced carrier scattering. Similarly, Tsujii and Mori [2] reported the same trend in Cu-deficient and Fe-excess chalcopyrite, suggesting that excess metal vacancies enhance the hole concentration while introducing additional scattering centers that reduce the mobility. In contrast, sulfur non-stoichiometry can have distinct effects. Li et al. [24] found that a S deficiency in chalcopyrite reduced both the carrier concentration and mobility, likely due to the formation of compensating donor-like defects, while Xie et al. [25] reported that excess sulfur led to a decrease in carrier concentration and an increase in mobility, possibly due to a reduction in ionized impurity scattering. However, it is important to note that chalcopyrite is an n-type semiconductor, where electrons are the majority charge carriers, whereas Cu_3+m_SbS_3_ remains a p-type regardless of the Cu content.

**Figure 6:**
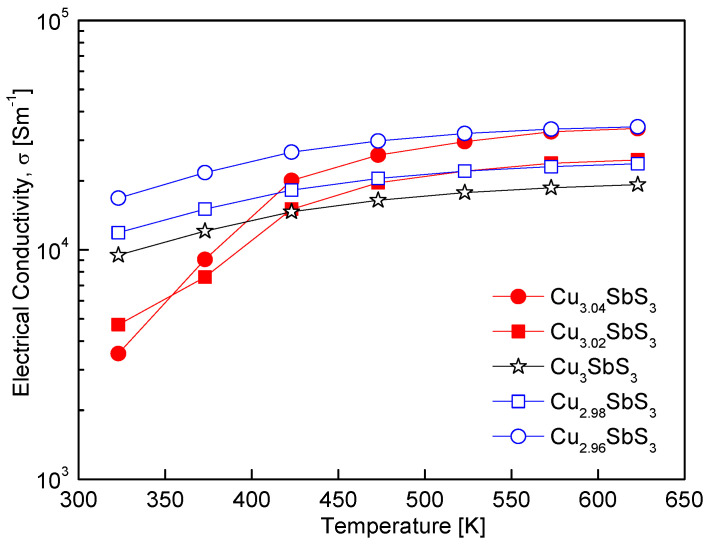
Temperature dependence of the electrical conductivity of Cu_3+m_SbS_3_.

Figure 6 shows the temperature-dependent electrical conductivity of the Cu_3+m_SbS_3_, demonstrating the characteristic behavior of non-degenerate semiconductors, where the conductivity increases with temperature due to thermally activated charge carriers. Within the 323–423 K range, the electrical conductivity varied based on the Cu non-stoichiometry, with the Cu-deficient and Cu-excess specimens exhibiting either enhanced or reduced conductivity compared to the stoichiometric skinnerite. However, above 423 K, all the non-stoichiometric specimens exhibited superior electrical conductivity relative to the stoichiometric sample. The Cu-excess specimens displayed a particularly strong temperature dependence, achieving significantly higher conductivity at elevated temperatures, which can be attributed to the formation of the tetrahedrite phase. Since tetrahedrite possesses an inherently high electrical conductivity, its presence likely contributed to the enhanced transport properties observed at higher temperatures.

Kim et al. [32] reported that tetrahedrite synthesized via the MA–HP process exhibited electrical conductivity ranging from (2.6–3.8) × 10^4^ Sm^−1^ over 323–723 K, surpassing the conductivity of the skinnerite in this study, which ranged from (1.0–1.9) × 10^4^ Sm^−1^ over 323–623 K. This suggests that the coexistence of skinnerite and tetrahedrite induces a complex charge transport mechanism, possibly due to phase boundary effects or variations in the carrier concentration and scattering. The interplay between these phases may enhance the electrical conductivity by providing additional conducting pathways, particularly at high temperatures where the contribution of tetrahedrite becomes more pronounced. Previous studies have demonstrated that even slight variations in the Cu content can significantly influence the electrical conductivity of Cu-based chalcogenides. For example, Kim and Kim [19] observed an increase in the electrical conductivity of Cu-deficient permingeatite, which was attributed to an increase in the carrier concentration due to the Cu vacancy formation. Similarly, Kwak and Kim [28] found that a Cu deficiency in tetrahedrite led to enhanced electrical conductivity, while a Cu excess resulted in a decline, likely due to increased carrier scattering and secondary-phase formation. These findings collectively indicate that the Cu non-stoichiometry is a crucial factor for tuning both the carrier concentration and electrical conductivity of Cu–chalcogenide thermoelectric materials.

**Figure 7:**
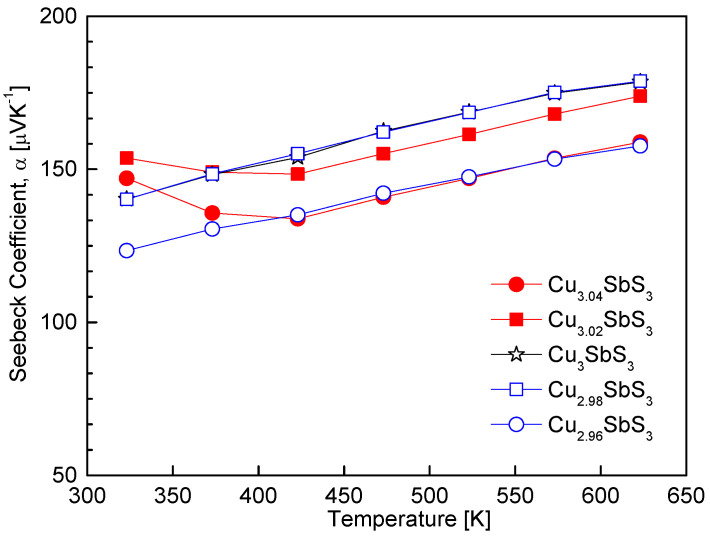
Temperature dependence of the Seebeck coefficient for Cu_3+m_SbS_3_.

Figure 7 presents the temperature dependence of the Seebeck coefficient for the Cu_3+m_SbS_3_, confirming the p-type conduction behavior of all the specimens through their positive Seebeck coefficients. The increase in the Seebeck coefficient with rising temperature suggests a thermally activated carrier transport mechanism, consistent with the behavior of non-degenerate semiconductors. Within the measured temperature range, no intrinsic transition was observed, indicating that the dominant charge carriers remained unchanged. For the stoichiometric skinnerite, the Seebeck coefficient ranged from 140 to 178 μV K^−1^ over 323–623 K. The variation in the Seebeck coefficient with respect to the Cu content exhibited an inverse correlation with the electrical conductivity, aligning with the well-established trade-off between these two parameters due to their mutual dependence on the carrier concentration.

The Cu-deficient specimens exhibited a reduced Seebeck coefficient, which can be attributed to the increase in the hole concentration caused by the Cu vacancy formation. As the Seebeck coefficient is inversely proportional to the carrier concentration according to the Pisarenko relation [1], the observed decrease in the Seebeck coefficient is consistent with previous studies on Cu-based chalcogenides. In contrast, the Cu-excess specimens exhibited an anomalous trend that deviated from the expected behavior. This deviation was likely due to the coexistence of the skinnerite and tetrahedrite phases, as discussed in Figure 6. Since tetrahedrite exhibits distinct charge transport characteristics, including a relatively high carrier concentration and moderate Seebeck coefficient, its presence could have led to complex carrier scattering mechanisms and energy filtering effects, ultimately influencing the measured Seebeck coefficient. Similar trends have been reported in previous studies, where an increase in the carrier concentration due to a Cu deficiency resulted in a reduction in the Seebeck coefficient for various Cu-based chalcogenide thermoelectric materials. For instance, a decrease in the Seebeck coefficient with an increasing carrier concentration has been observed in famatinite [17], permingeatite [18,19,20], chalcopyrite [22,23], and tetrahedrite [28].

**Figure 8:**
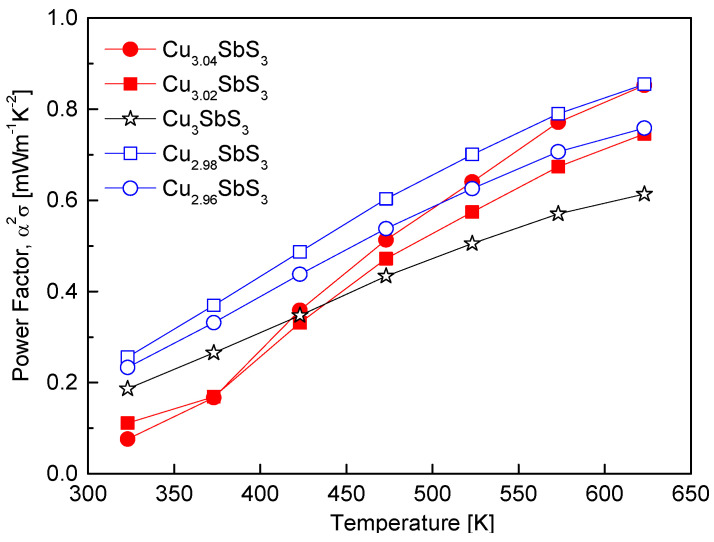
Temperature dependence of the power factor for Cu_3+m_SbS_3_.

Figure 8 illustrates the variation in the power factor of the Cu_3+m_SbS_3_, which is determined by the interplay between the Seebeck coefficient and the electrical conductivity. Since the electrical conductivity is proportional to the carrier concentration, whereas the Seebeck coefficient exhibits an inverse relationship with the carrier concentration, optimizing the carrier concentration is crucial for achieving a high power factor [34]. In this study, Cu_2.98_SbS_3_ exhibited the highest power factor of 0.85 mWm^−1^K^−2^ at 623 K, which is a significant improvement compared to the stoichiometric Cu_3_SbS_3_, which recorded a power factor of 0.61 mWm^−1^K^−2^ at the same temperature. This enhancement is primarily attributed to the Cu deficiency, which increased the electrical conductivity and, consequently, the power factor. The reduction in Cu^+^ ions due to the Cu deficiency promoted electron injection, thereby reinforcing the n-type conduction and increasing the electrical conductivity. This observation aligns with the findings of Wei et al. [20], who reported that Cu-deficient permingeatite (Cu_2.950_SbSe_4_ and Cu_2.925_SbSe_4_) exhibited power factors over 60% higher than their stoichiometric counterparts.

In contrast, the Cu-excess specimens displayed lower power factors at temperatures below 423 K, but exhibited an increasing trend above 423 K. This improvement at elevated temperatures is attributed to the formation of the tetrahedrite phase, which has been reported to achieve a high power factor of approximately 1 mWm^−1^K^−2^ at 623 K [32]. In this study, the presence of the tetrahedrite phase in the Cu-excess samples likely contributed to the enhancement in electrical conductivity at higher temperatures. These findings are consistent with previous studies on off-stoichiometric Cu–chalcogenide compounds. Li et al. [24] reported that S-deficient chalcopyrite (CuFeS_1.8_) exhibited a power factor approximately five times higher than stoichiometric CuFeS_2_. Similarly, Kwak and Kim [28] demonstrated that, in off-stoichiometric tetrahedrite (Cu_12+m_Sb_4_S_13_), Cu-deficient specimens exhibited higher power factors than Cu-rich specimens.

**Figure 9:**
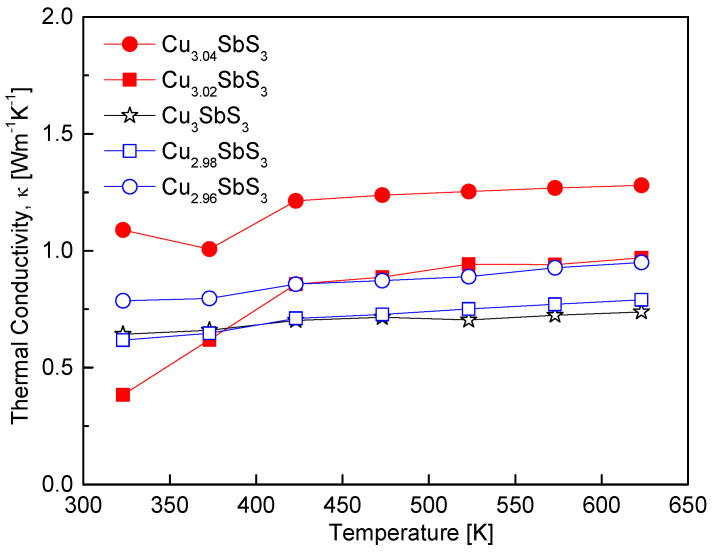
Temperature dependence of the thermal conductivity for Cu_3+m_SbS_3_.

Figure 9 presents the temperature dependence of the thermal conductivity of the Cu_3+m_SbS_3_. The thermal conductivity of all the specimens exhibited minimal variation with the temperature, indicating that phonon scattering mechanisms remained relatively stable across the measured range. However, distinct differences in the thermal conductivity were observed depending on the Cu content, particularly below 423 K, similar to the trends observed for the other thermoelectric parameters, such as the electrical conductivity and the Seebeck coefficient. For the stoichiometric Cu_3_SbS_3_, the thermal conductivity ranged from 0.62 to 0.72 Wm^−1^K^−1^ between 323 and 623 K. This behavior is comparable to the values reported by Zhang et al. [7] for cubic skinnerite (0.45–0.70 Wm^−1^K^−1^) over a similar temperature range, confirming the presence of the cubic skinnerite phase in the synthesized specimens. The Cu-deficient specimens exhibited a slight increase in thermal conductivity, which can be attributed to the rise in the carrier concentration. In contrast, the Cu-excess specimens displayed significantly higher thermal conductivity values, likely due to the formation of tetrahedrite as a secondary phase. The presence of tetrahedrite is known to influence the thermal transport properties, as reported by Du et al. [12], who observed that orthorhombic skinnerite exhibited lower thermal conductivity (0.30–0.25 Wm^−1^K^−1^) compared to tetrahedrite (0.85–0.95 Wm^−1^K^−1^) over the same temperature range. The relatively higher thermal conductivity of tetrahedrite arises from its metallic-like electronic transport. Consequently, the formation of tetrahedrite in the Cu-excess specimens led to a notable increase in the overall thermal conductivity, particularly at higher temperatures.

**Figure 10:**
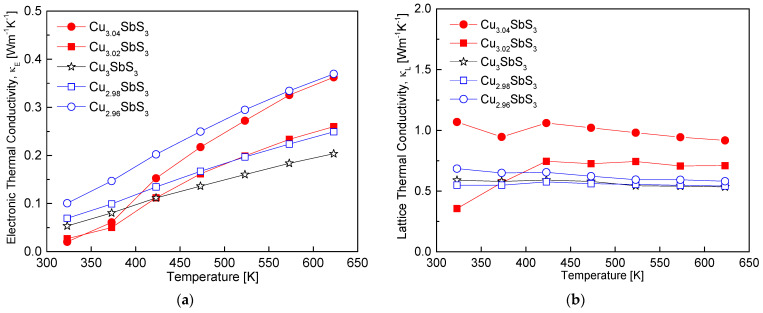
Separated thermal conductivity of Cu_3+m_SbS_3_: (**a**) electronic thermal conductivity and (**b**) lattice thermal conductivity.

Figure 10 presents the decomposition of the total thermal conductivity of the Cu_3+m_SbS_3_ into its electronic (κ_E_) and lattice (κ_L_) components. As outlined in the introduction, the total thermal conductivity comprises contributions from the charge carriers and phonon transport [35]. The electronic thermal conductivity was estimated using the Wiedemann–Franz law (κ_E_ = LσT), where the Lorenz number (L) was calculated using the empirical expression L = 1.5 + exp(−|α|/116) [36]. As shown in Figure 10a, the variation in the κ_E_ with the Cu composition and temperature closely follows the electrical conductivity trend observed in Figure 6, as both parameters are directly related to the charge carrier transport. The stoichiometric Cu_3_SbS_3_ exhibited κ_E_ values ranging from 0.05 to 0.20 Wm^−1^K^−1^ over 323–623 K, indicating that deviations from the stoichiometry increased the electronic contribution to the thermal conductivity. This increase can be attributed to the enhanced carrier concentration in the Cu-deficient specimens and the formation of conductive tetrahedrite phases in the Cu-excess specimens, both of which contributed to an increase in the κ_E_.

Figure 10b presents the lattice thermal conductivity (κ_L_), which follows a trend similar to that of the total thermal conductivity shown in Figure 9. This observation suggests that phonon transport dominates the thermal conduction mechanism in skinnerite. The stoichiometric Cu_3_SbS_3_ sample exhibited relatively low κ_L_ values, ranging from 0.53 to 0.59 Wm^−1^K^−1^ over 323–623 K, which is comparable to the reported values for cubic skinnerite. In contrast to tetrahedrite, where a Cu excess enhances phonon scattering and reduces the κ_l_ due to increased structural disorder and rattling effects [28], and eskebornite, where Cu_2_Se secondary phases and Se vacancies serve as effective phonon scattering centers [26], the introduction of non-stoichiometry in the skinnerite resulted in an increase in the lattice thermal conductivity. Despite expectations that a Cu deficiency would induce vacancy formation and that a Cu excess would introduce solid solution atoms capable of enhancing phonon scattering, these mechanisms were not sufficiently effective at reducing the lattice thermal conductivity of skinnerite. This suggests that the dominant phonon scattering mechanisms in skinnerite are less sensitive to compositional deviations than in other related thermoelectric materials, possibly due to its inherently low anharmonicity and reduced lattice disorder.

**Figure 11:**
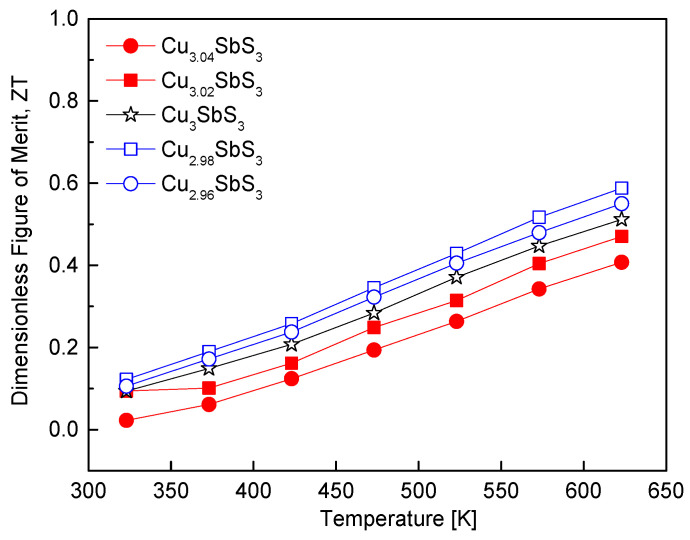
Dimensionless figure of merit for Cu_3+m_SbS_3_.

Figure 11 illustrates the temperature-dependent ZT values of the Cu_3+m_SbS_3_. The ZT is a key performance metric for thermoelectric materials and is defined as the ratio of the power factor to the thermal conductivity at the application temperature. In this study, although the thermal conductivity increased with the temperature, the concurrent enhancement in the power factor resulted in an overall improvement in the ZT. Among the synthesized compositions, the Cu-deficient Cu_2.98_SbS_3_ exhibited the highest power factor, leading to a peak ZT of 0.59 at 623 K. This value surpassed that of the stoichiometric skinnerite (ZT = 0.51 at 623 K), highlighting the beneficial effect of a Cu deficiency for optimizing the charge transport properties. The improvement in the ZT can be primarily attributed to the increase in the electrical conductivity associated with the optimized carrier concentration, while maintaining a relatively low thermal conductivity. In contrast, the Cu-excess specimens did not show a significant improvement in the ZT. Despite an increase in the power factor, the concurrent rise in the thermal conductivity due to the formation of tetrahedrite limited any further enhancement in the thermoelectric performance. This suggests that the introduction of a Cu excess not only increases the carrier concentration but also enhances the lattice contribution to the thermal conductivity, offsetting the benefits of improved electrical transport.

These observations align with previous studies on Cu-based chalcogenides. Zhang et al. [7] reported that stoichiometric Cu_3_SbS_3_ synthesized via mechanical alloying, hot pressing, annealing, and quenching achieved a ZT of 0.57 at 623 K. Similarly, Wei et al. [20] found that stoichiometric permingeatite Cu_3_SbSe_4_ exhibited a ZT of 0.2 at 673 K, whereas Cu-deficient Cu_2.925_SbSe_4_ reached a maximum ZT of 0.50 at 673 K, demonstrating the beneficial effects of a Cu deficiency on the thermoelectric performance. A similar trend has been observed in other Cu–chalcogenide systems. Chen et al. [27] reported that Sb-deficient CuSb_0.91_Se_2_ in eskebornite exhibited a maximum ZT of 0.50 at 673 K, attributed to improved electrical properties and reduced thermal conductivity. Kwak and Kim [28] observed that stoichiometric tetrahedrite Cu_12_Sb_4_S_13_ exhibited a ZT of 0.86 at 723 K, while Cu-deficient Cu_11.9_Sb_4_S_13_ achieved a maximum ZT of 0.91 at 723 K. These results collectively underscore the critical role of non-stoichiometry in tuning the electrical and thermal properties of Cu-based chalcogenides, demonstrating that precise compositional control is essential for optimizing the thermoelectric performance.

## 4. Conclusions

Non-stoichiometric skinnerite compounds, Cu_3+m_SbS_3_ (−0.04 ≤ m ≤ 0.04), were synthesized via mechanical alloying followed by hot pressing. The phase evolution, microstructural characteristics, charge transport behavior, and thermoelectric properties were systematically investigated as a function of the Cu composition. A Rietveld refinement of the X-ray diffraction data, combined with scanning electron microscopy and energy-dispersive X-ray spectroscopy, confirmed that the primary phase was cubic skinnerite, whereas the Cu-excess specimens exhibited the formation of a secondary cubic tetrahedrite phase. The Seebeck coefficient exhibited a positive temperature dependence, with no discernible intrinsic transition observed within the investigated temperature range. Due to the inverse relationship between the Seebeck coefficient and electrical conductivity (or carrier concentration), the Seebeck coefficient followed an opposing trend to that of the electrical conductivity. The enhancement in the power factor was primarily attributed to an increase in the carrier concentration induced by the Cu deficiency, as well as the presence of the secondary tetrahedrite phase in the Cu-excess specimens, both of which contributed to the improved charge transport properties. The thermal conductivity exhibited minimal temperature dependence, with the electronic contribution scaling with the carrier concentration and electrical conductivity, while the lattice thermal conductivity remained the dominant component. Despite the overall increase in the total thermal conductivity with temperature, the concurrent enhancement in the power factor resulted in an improvement in the thermoelectric figure of merit. Among the compositions investigated, Cu_2.98_SbS_3_ exhibited the highest power factor of 0.85 mWm^−1^K^−2^ and achieved a maximum ZT of 0.59 at 623 K, demonstrating the effectiveness of a Cu deficiency for optimizing the thermoelectric performance of skinnerite-based materials.

## Figures and Tables

**Table 1:** Lattice constants, relative densities, and phase contents of Cu_3+m_SbS_3_ prepared using the MA–HP method.

Specimen	Lattice Constant *a* [nm]	Relative Density [%]	Phase Content [wt.%]	
			Skinnerite	Tetrahedrite
Cu_3.04_SbS_3_	1.0341	99.1	53.3	46.7
Cu_3.02_SbS_3_	1.3041	98.8	72.8	27.2
Cu_3_SbS_3_	1.0374	99.7	100.0	0.0
Cu_2.98_SbS_3_	1.0346	99.0	100.0	0.0
Cu_2.96_SbS_3_	1.0347	98.2	100.0	0.0

## Data Availability

The original contributions presented in this study are included in this article, further inquiries can be directed to the corresponding author.
